# *Streptomyces* from traditional medicine: sources of new innovations in antibiotic discovery

**DOI:** 10.1099/jmm.0.001232

**Published:** 2020-07-21

**Authors:** Gerry A. Quinn, Aiya M. Banat, Alyaa M. Abdelhameed, Ibrahim M. Banat

**Affiliations:** ^1^​ Centre for Molecular Biosciences, Ulster University, Coleraine, Northern Ireland, UK; ^2^​ Department of Orthopaedics, Altnagelvin Hospital, Londonderry, Northern Ireland, UK; ^3^​ Department of Biotechnology, College of Science, University of Diyala, Baqubah, Iraq

**Keywords:** ethnopharmacology, extreme environments, endophytes, pathogens, secondary metabolites, silent gene clusters

## Abstract

Given the increased reporting of multi-resistant bacteria and the shortage of newly approved medicines, researchers have been looking towards extreme and unusual environments as a new source of antibiotics. *Streptomyces* currently provides many of the world’s clinical antibiotics, so it comes as no surprise that these bacteria have recently been isolated from traditional medicine. Given the wide array of traditional medicines, it is hoped that these discoveries can provide the much sought after core structure diversity that will be required of a new generation of antibiotics. This review discusses the contribution of *Streptomyces* to antibiotics and the potential of newly discovered species in traditional medicine. We also explore how knowledge of traditional medicines can aid current initiatives in sourcing new and chemically diverse antibiotics.

## Introduction

*Streptomyces* are the source of many of the world’s antibiotics and in this respect they represent a very important bacterial genus [1]. They are present in almost all environments from deep sea to high mountains [2–4]. They are Gram-positive, filamentous, spore-forming bacteria that are members of the phylum *Actinobacteria *. *Streptomyces* diverged from their closest relative, *Kitasatospora,* approximately 382 million years ago in the late Devonian period, coinciding with the appearance of land vertebrates [5, 6]. *Streptomyces* are non-motile bacteria that spread by producing threadlike hyphae which penetrate surfaces in search of nutrients. When resources are limited, *Streptomyces* produce aerial hyphae that divide, producing spores that can resist unfavourable conditions and are easily dispersed to new environments or sources of nutrients [7].

During this growth phase *Streptomyces* produce secondary metabolites: compounds that are not strictly necessary for growth or reproduction, but can give the organism a competitive advantage [7]. These metabolites help the vegetative bacterial cells by sequestering metals such as iron (siderophores), protecting them from UV light (through pigmentation), inhibiting competitors (antibiotics) and also facilitating communication with other species [8]. This molecular diversity is possible in *Streptomyces* through their comparatively large genome, which can be quadruple the size of some other bacterial genomes [7]. In this review, we look at the contribution of *Streptomyces* to antimicrobial chemotherapy, new innovations in bioprospecting through the association of *Streptomyces* with traditional medicine and the application of this knowledge to antibiotic discovery.

### Antibiotics from *Streptomyces*

The antibiotic streptomycin was discovered in 1943 by Albert Schatz, a PhD student of Selman Waksman, with help from others including Doris Ralston, Elizabeth Bugie and Christine Reilly [9]. During World War II, there was an urgent drive to find antibiotics that could fill the gap left by penicillin, which was ineffective against tuberculosis (TB) and some Gram-negative pathogens. Inspired by Fleming’s discovery, Waksman instructed his PhD student to screen bacterial isolates against a highly virulent TB strain in the basement of his laboratory. It was here that Schatz discovered streptomycin from an isolate of *Streptomyces griseus* originating from heavily manured compost soil and another from a chicken gizzard [9]. Given the importance of such a discovery, the first vial of streptomycin, which Schatz presented to his mother, is still on display in the Smithsonian Institution [10]. Selman Waksman used his drug company connections to conduct the large-scale trials necessary to prove that streptomycin was effective against TB, bubonic plague, typhoid fever and cholera [2, 9, 11, 12]. Waksman was credited with coining the term ‘antibiotic’ winning the Nobel Prize for Medicine in 1952 and patenting eight antibiotics [4], some of which are detailed in Table 1.

**Table 1.** Clinically and economically important bioactive molecules from *Streptomyces* species, including name, mode of action and source

Bioactive molecule	Type	Species	Location /soil type
Bialaphos	Herbicide	* S. hygroscopicus *	Easter Island, soil [74]
Bleomycin	Anticancer	'*S. verticillus*'	Soil, coal mine [75]
Chloramphenicol	Antibiotic	* S. venezuelae *	Soil and compost [76]
Cineromycin A+B	Inhibits adipocyte differentiation of 3T3-L1 cells via Krüppel-like factors 2 and 3	* S. cinerochromogenes *	Tama Graveyard soil, Tokyo, Japan [77]
Clavulanic acid	β-lactamase inhibitor	* S. clavuligerus *	South American soil sample [78]
Clindamycin + lincomycin	Antibiotic + Antibiotic for mycoplasmas and *Actinomyces *	* S. lincolnensis *	Lincoln, NE, USA [79]
Daptomycin	Lipopeptide antibiotic	* S. roseosporus *	Mount Ararat, Turkey [80]
Erythromycin	Antibiotic	* S. erythraeus *	Soil, Philippines [81]
Fosfomycin	Broad-spectrum antibiotic against urinary tract infections	* S. fradiae *	Soil, Mount Montgo, Spain [82]
Ivermectin	Antiparasitic, anti-onchocerciasis and anti-lymphatic filariasis	* S. avermitilis *	Japanese golf course [83]
Kanamycin	Antibiotic	* S. kanamyceticus *	Soil, Nagano, Japan [84]
Neomycin	Antibiotic	* S. fradiae* and '*S. albogriseus*'	Soil [85]
Nystatin	Antifungal	* S. noursei *	Garden soil [86]
Rapamycin	Antifungal, antitumour immunosuppressive	* S. hygroscopicus *	Easter Island, soil [87]
Saframycin(s) A, B, C, D and E	Anticancer	* S. lavendulae* subsp. *grasserius *	Tama Graveyard, Tokyo, Japan [88]
Streptomycin	Antibiotic against TB, cholera, bubonic plague	* S. griseus *	Compost manure, Rutgers Farm, New York, USA [12]
Tetracycline	Antibiotic	* S. aureofaciens* and *S. rimosus *	Timothy grassland, Sanborn Field, University of Missouri, USA [89]
Vancomycin	Antibiotic	*'S. orientalis'* (now named *Amycolatopsis orientalis *)	Borneo dirt [90]

Up until the 1970s, it was still relatively easy to isolate new compounds from *Streptomyces,* but since 1985 only three new classes of antibiotics that have been discovered [13–15]. One of these compounds is platensimycin, a new class of antibiotic from *Streptomyces platensis* that selectively inhibits cellular lipid biosynthesis. This was discovered by the Merck group [13, 14].

Very recently, an antibiotic-producing strain of thermotolerant *Streptomyces* sp. TM32 was isolated from the rhizosphere of *Curcuma longa* L., a medicinal plant [16]. This is believed to be a new strain of *Streptomyces sioyaensis* that has strong antimicrobial activities against both human and plant pathogens, including an antibiotic-resistant pathogen, *Staphylococcus haemolyticus* MR-CoNS [16]. It may also serve as an emerging source for further discovery of valuable and novel bioactive compounds.

### Antibiotic mode of action

There are a few common modes of action for *Streptomyces* antibiotics targeting cellular components of bacteria. The first discovered mechanism was the interference with bacterial protein synthesis by blocking ribosomal functional sites [17]. Tetracycline, streptomycin, kanamycin and gentamicin block protein synthesis by binding to the small ribosomal subunit (30S), whereas erythromycin, clindamycin and chloramphenicol target the large ribosomal subunit (50S). Ciprofloxacin and novobiocin interfere with bacterial DNA translation. Carbapenems, cephalosporins, vancomycin, fosfomycin, bacitracin and daptomycin, in comparison, interfere with the bacterial cell wall or cell-membrane integrity and synthesis [18, 19].

### Antibiotic synthesis

*Streptomyces* generally synthesize their antibiotics using large enzymatic complexes like polyketide synthases (PKSs), non-ribosomal peptide synthases (NRPSs) or a combination of both. These large multienzyme complexes use many different domains to accomplish chemical modifications that can produce a wide range of antibiotics [20, 21].

**Fig. 1.**
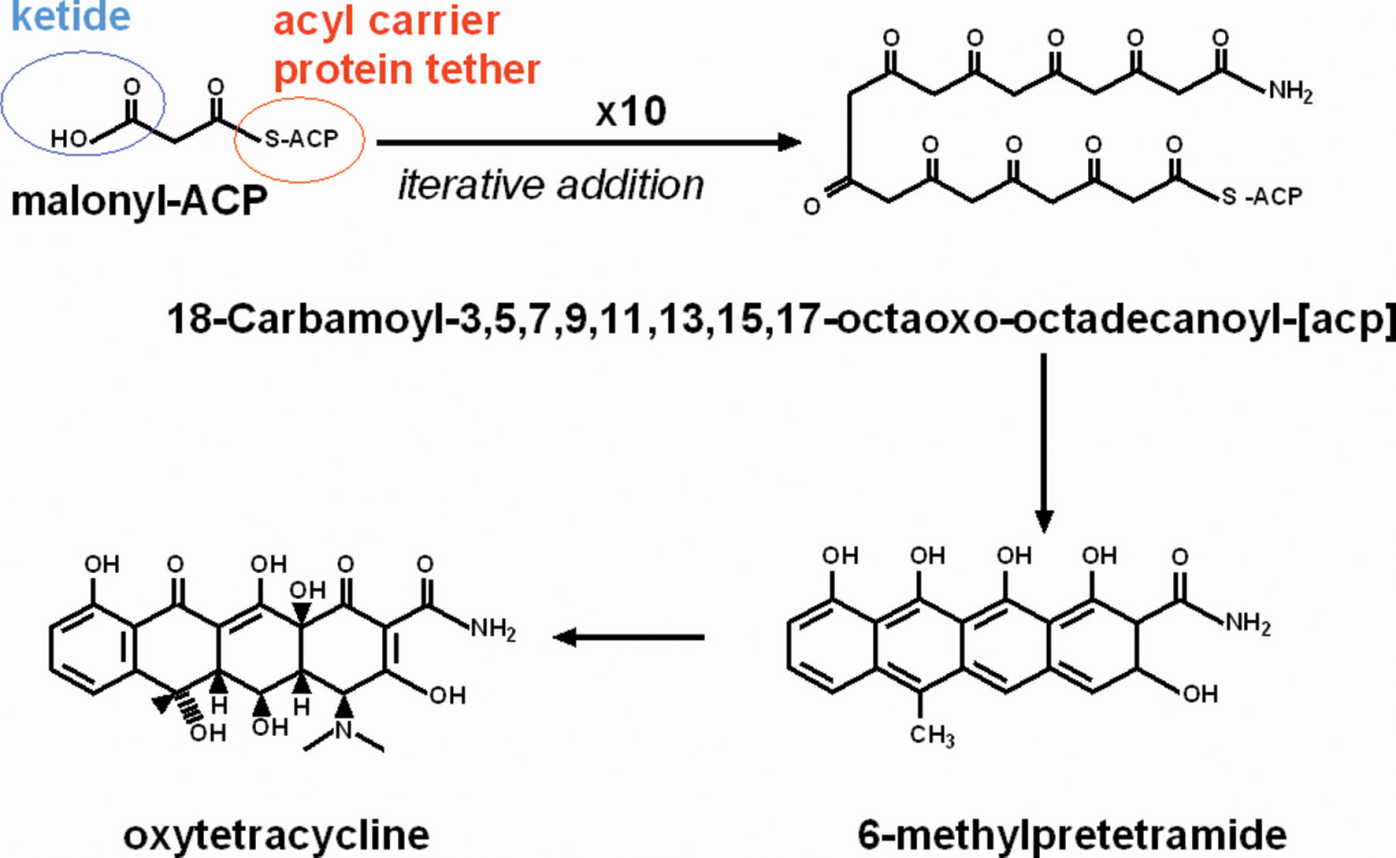
Oxytetacyline synthesis by the PKS type II system: consecutive modules of the PKS type II enzyme catalyse the successive decarboxylative condensations of malonyl CoA, followed by modifications by transferases, oxygenases and cyclases, and additional modifications to produce oxytetracycline. Figure adapted from that of the Nomenclature Committee of the International Union of Biochemistry and Molecular Biology (NC-IUBMB) in consultation with the IUPAC-IUBMB Joint Commission on Biochemical Nomenclature (JCBN) [91].

In the PKS system, antibiotics typically begin as a ketide monomer attached to an acyl carrier protein. Construction of the antibiotic proceeds through a series of enzyme-mediated steps typically involving acyltransferases, ketidesynthases and other enzymes leading to the formation of the backbone of the polyketide antibiotic. The growing antibiotic can be subject to further modifications that might include cyclization, decarboxylation, dehydration, reduction and methylation [21] (Fig. 1).

**Fig. 2.**
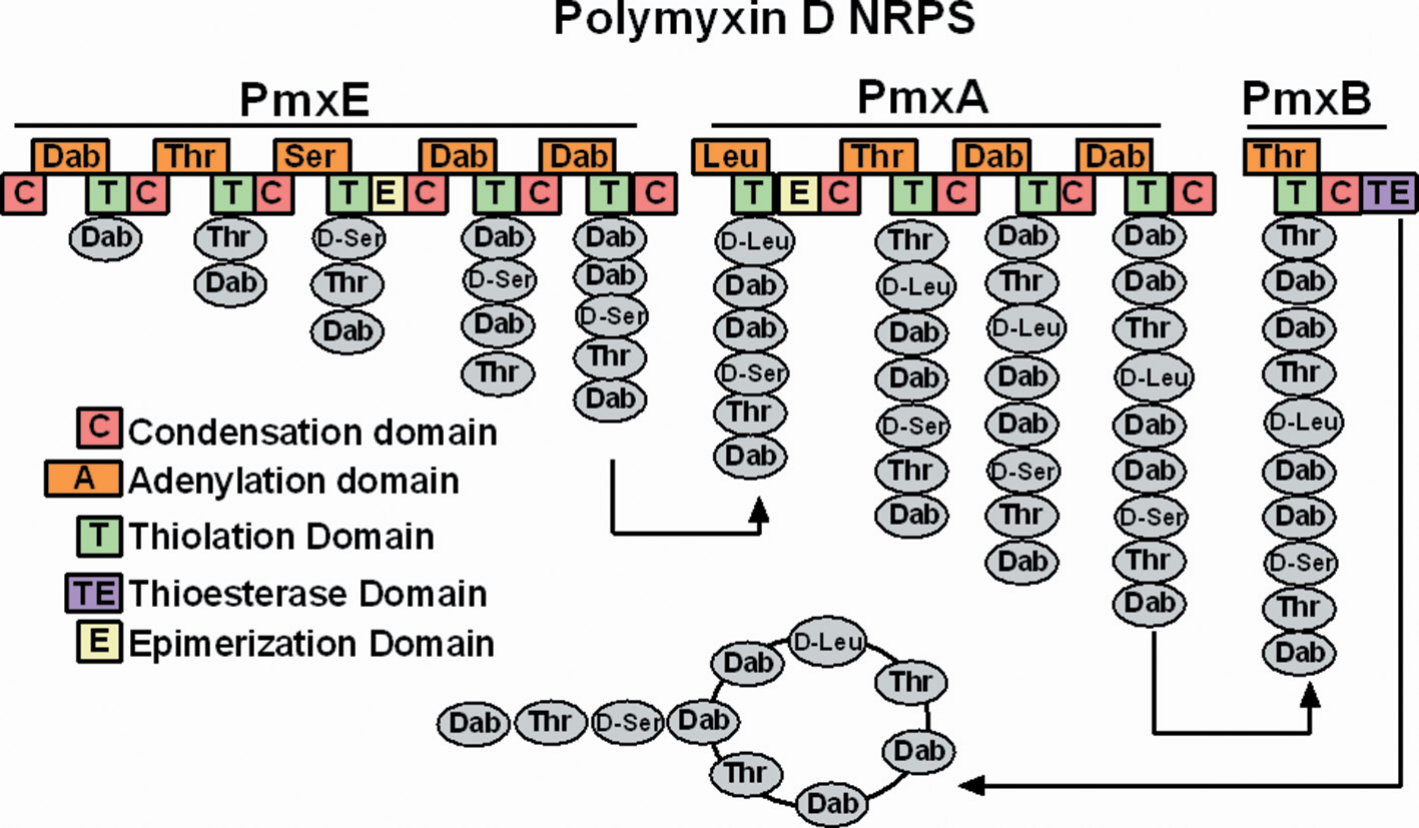
NRPS system for polymyxin from *Paenibacillus polymyxa *. The NRPS enzyme is composed of many modules that contain subunits (boxes) that help to attach amino acids. The thioesterase domain (TE) is responsible for cyclizing and releasing the peptide at the end of synthesis. Figure reproduced by kind permission of Dr T. Velkov [92].

Non-ribosomal peptide synthesis is carried out by large enzymatic complexes. These enzymes can be found in many types of bacteria and are organized in modules responsible for the addition of one amino acid at a time [20] (Fig. 2). NRPS peptides may also contain nonstandard amino acids such as diaminobutyric acid (Dab), and can be modified by glycosylation, amidation and halogenation amongst other processes [20].

### Discovery of *Streptomyces* in traditional medicine

Traditional medicine containing antibiotics has been around and used in local remedies for millennia without knowledge of its active principles. One of the earliest connections between *Streptomyces* and traditional medicine is the Red Soil of Jordan, which has been used as a cure for skin infections for millennia [22]. More definitive connections have been found in Africa, where researchers discovered that some ancient Nubian bones (~300 AD) contained tetracycline. This was traced back to a local beer drunk by the Nubians containing oats that had *Streptomyces* growing on them [23].

Following the UK Medical Act (1852), traditional medical practitioners who were not officially recognized were prohibited from claiming to cure illnesses. This saw traditional medicine in the UK fade into the background apart from in remote rural areas [24]. It would then be another 80 years before antibiotics made an official appearance in clinical practice with the discovery of penicillin [25].

### *Streptomyces* from traditional medicinal plants

One of the inspirations for research into traditional medicine may have come from Geoffrey Cordell, who devised a series of systematic searches of plant metabolites for anticancer medicines. This included an ethno-medical approach, which evaluated written or historical evidence from traditional medicinal practice [26]. Since then, many *Streptomyces* have been isolated from traditional plant medicines, especially in areas of low nutrient availability or extreme physiological conditions [27, 28]. These *Streptomyces* can exist as endophytes that live at least a part of their life cycle inside plant tissues without causing damage, or epiphytes that live on the outside of the plants [29, 30]. *Proteobacteria* and *Actinobacteria* have been reported as the most frequent endophytic species [31]. Many studies focused on the largest areas of traditional medicine such as China and India, but there have also been discoveries in South America and Africa [32, 33] (Table 2).

**Table 2.** *
Streptomyces* spp. isolated from traditional medical plants, including source, type and location

Plant	Antibiotic	Location	* Streptomyces *	Reference
*Arnica montana* L., wolf’s bane	Cycloheximide, actiphenol, diketopiperazine	Mountain Nutrient-poor soil, Brazil	* Streptomyces* spp., strong antifungal	[64]
*Paraboea sinensis*	Vinaceuline cyclodipeptides	Rocks and cliffs, Vietnam	* Streptomyces* sp. YIM 64018	[93]
*Lychnophora ericoides* Mart.	2,3-dihydro-2,2-dimethyl-4(1H)-quinazolinone, nocardamine	Brazil	Effective against *Trypanosoma cruzi*	[64]
*Achnatherum inebrians*, Drunken Horse Grass	Whole extracts	Mountain Xinjiang, China	* Streptomyces albus * effective against *Aphis gossypii*	[94]
*Dracaena cochinchinensis* Lour., Dragons blood	Actinomycin-D, novel SPE-B11.8	Ninh Binh province, Vietnam	* Streptomyces* sp. HUST012, effective against MRSA, MRSE, *Escherichia coli* and *Klebsiella pneumoniae *	[95]
*Vochysia divergens*	Brevianamide F and cyclo-(l-Pro-l-Phe)	Brazil wetlands Pantanal	Effective against MRSA	[96]
*Heracleum souliei*	Pluramycin	China	* Streptomyces* sp. Y3111	[31]

*Staphylococcus epidermidis (MRSE),* usually harmless skin commensal that can cause difficult-to-treat multi-resistant infections.

### *Streptomyces *-linked traditional medicine from invertebrates

**Fig. 3.**
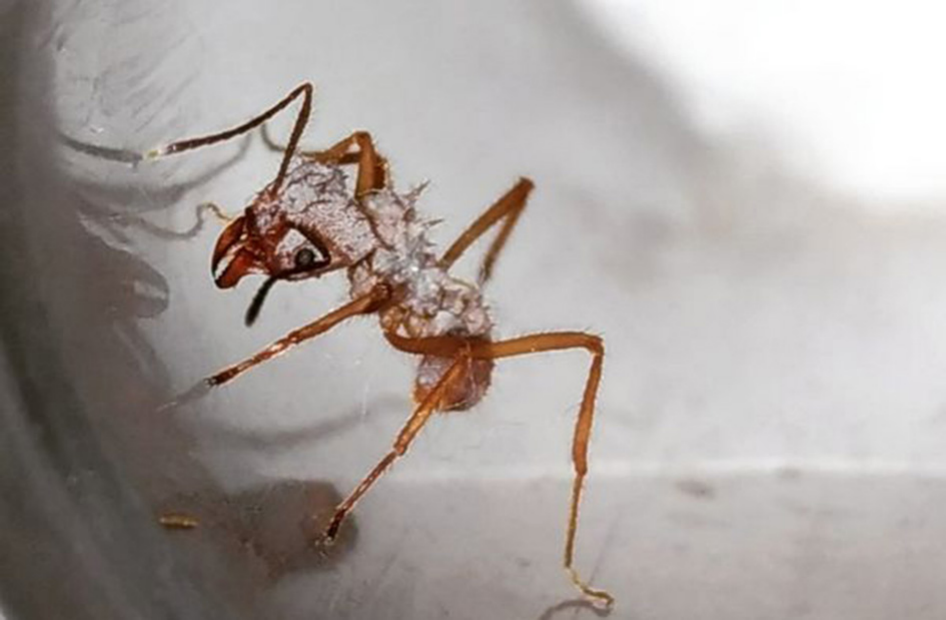
A leafcutter ant (*Acromyrmex*) covered in *Pseudonocardia *. Photograph by João Pedro Sá Medeiros (Antwiki – https://www.antwiki.org/wiki/Acromyrmex) [40].

Invertebrates have many associations with *Streptomyces* and traditional medicine [34–36]. In northern India, a paste made from crushed black ants (*Bothroponera rufipes*) has been reported to be used to treat scabies, wounds and boils [37]. Additionally, ground ants mixed with water are used to relieve toothaches [34]. What makes these remedies so interesting is that *Streptomyces* are associated with certain parts of the ant's exoskeleton [38]. In some cases, the ants rub their legs over these patches (of *Streptomyces *) and then onto areas where they farm fungi [38, 39]. A prime example of this is leafcutter ants (*Acromyrmex*), which use a species of *Actinomycete* known as *Pseudonocardia* as a defence against invasive parasites in their fungal gardens [40] (Fig. 3).

**Fig. 4.**
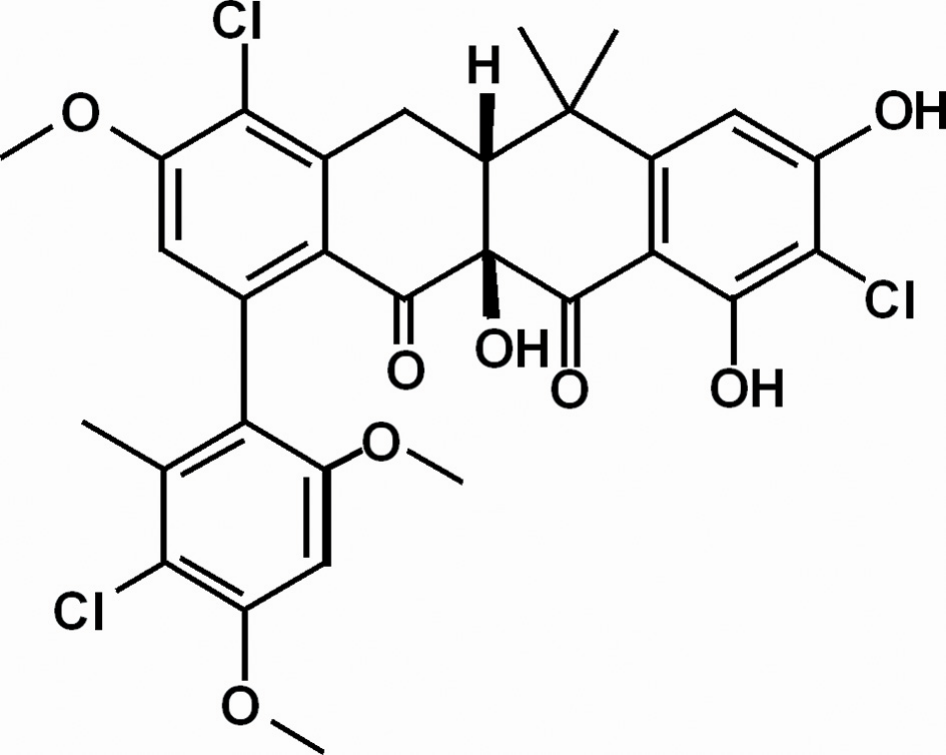
Formicamycin E. Adapted from [38].

It has also been found that African *Tetraponera penzigi* ants living in hollows inside Acacia trees called domatia harbour *Streptomyces formicae *, which produces potent antibiotics known as formicamycins (Fig. 4). These antibiotics have been reported to have inhibitory effects against meticillin-resistant *Staphylococcus aureus* (MRSA) and vancomycin-resistant enterococci [38].

The interactions between *Streptomyces* and ants has prompted researchers to examine other symbiotic relationships in invertebrates. For instance, it was recently discovered that mud dauber wasps had an association with *Streptomyces* that produce a novel polyunsaturated and polyoxygenated macrocyclic lactam (sceliphrolactam) which is an antifungal agent [41].

### Sponges

Sponges have a very long history in traditional medicine [42], although their symbiotic relationships with antibiotic-producing *Streptomyces* has only recently been discovered [43]. It is thought that *Streptomyces* from the marine environment can offer a potentially novel source of antimicrobial compounds [43, 44]. Chinese researchers recently reported the isolation of *Streptomyces tirandamycinicus* sp. nov., from a marine sponge with antibacterial potential against *Streptococcus agalactiae* [45]. Other researchers have isolated new compounds from *Streptomyces* associated with marine sponges in the Vietnam Sea, some of which are completely novel compounds active against both Gram-positive and Gram-negative bacteria and *Mycobacterium tuberculosis* [46]. Under certain conditions, these new bioactive compounds can also inhibit bacterial biofilm formation [47].

### Antibiotics from caves

Over the last few decades, karst and cave environments have become popular areas for bioprospecting of antimicrobial *Streptomyces* [48, 49]. Many of these areas are often associated with traditional medicine. Ancient texts suggest that a milky white exudate covering the surfaces of some caves called ‘*moonmilk*’ can heal multiple ailments [50]. Digging deeper, research has shown that moonmilk contains an abundance of *Streptomyces* that have antibacterial activity against a wide range of bacteria and fungi [49, 50], and display strong growth suppression against multi-resistant *Rasamsonia argillacea*, a causative agent of invasive mycosis in cystic fibrosis and chronic granulomatous diseases [50]. Similar studies from the Hampoeil cave (dolomite with limestone) in Iran, linked to Palaeolithic habitation, revealed many antimicrobial-producing *Streptomyces *, as well as other species [51].

### *Streptomyces* from traditional soil-based medicine

**Fig. 5.**
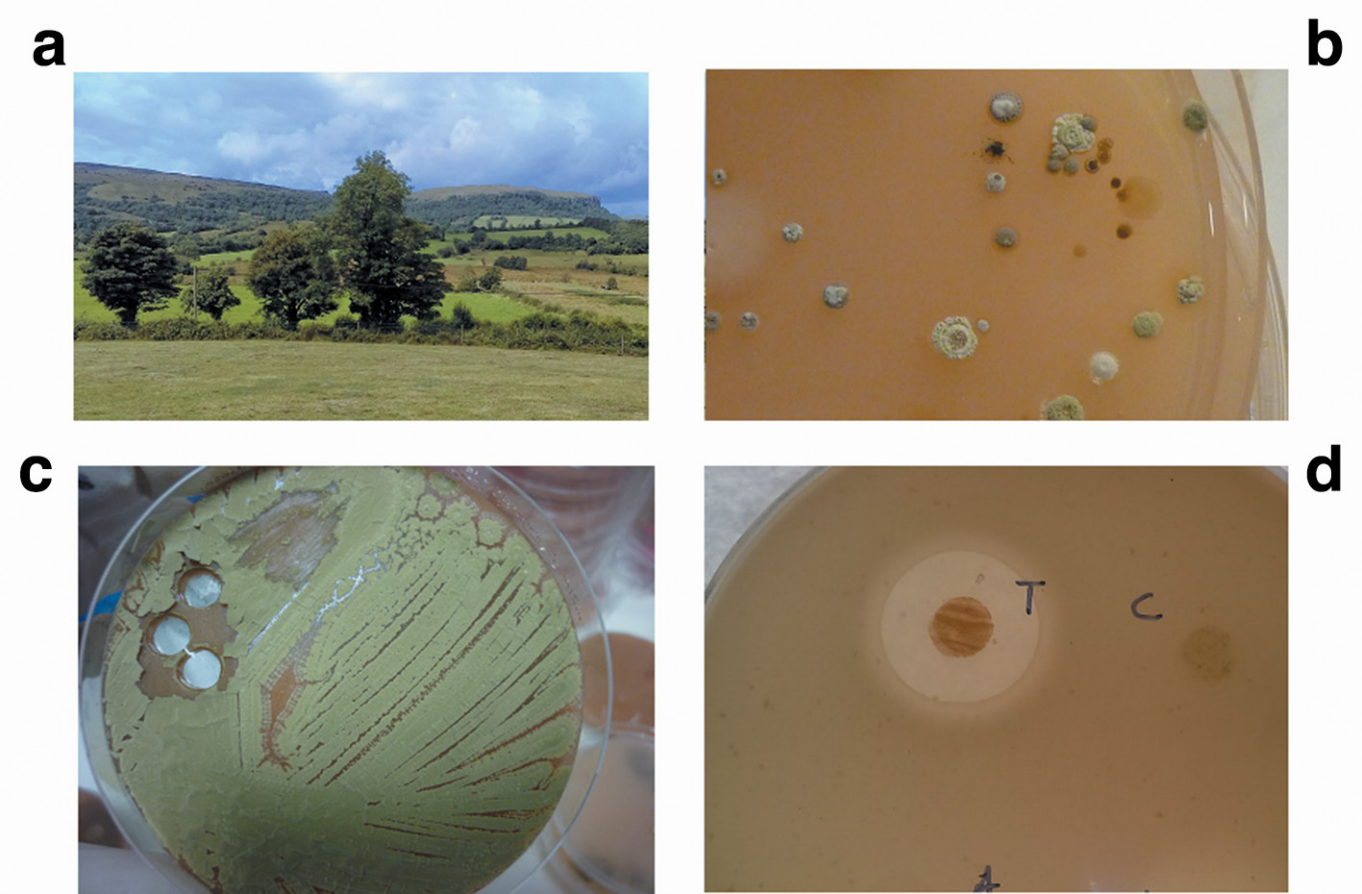
*
Streptomyces* from a traditional soil cure in the West Fermanagh Scarplands (a) was cultivated on selective isolation agar (b) yielding *Streptomyces* sp. myrophorea (c), which inhibited MRSA, as evidenced by a clear zone of inhibition (d).

The majority of *modern* antibiotics are derived from soil-based *Streptomyces *, so it is no surprise to discover that this media also features prominently in traditional medicine [22, 52]. Unlike *Streptomyces* discovered from the golden age of antibiotic discovery in the mid-20th century, traditional medicine soils are specific in their locations such as the Boho clay, or in their type such as glacial clay from Canada [52, 53]. Clay, which has long been thought to be therapeutic in itself, is also home to a diverse array of *Streptomyces* [22, 52–54]. Traditional glacial clay from Kisameet Bay in Canada has been used for millennia by the Heiltsuk people against skin infections [52]. When tested under laboratory conditions, this soil was shown to inhibit the growth of all six ESKAPE pathogens (* Enterococcus faecium *, *Staphylococcus aureus *, *Klebsiella pneumoniae *, *Acinetobacter baumannii *, *Pseudomonas aeruginosa* and *Enterobacter* spp.) [52]. On the other side of the Atlantic, researchers in Northern Ireland isolated a new species of *Streptomyces* from an ancient soil remedy in a region known as Boho, West Fermanagh [53]. This alkaline soil, lying on top of carboniferous limestone, contained a new species, *Streptomyces* sp. myrophorea, that inhibited several strains of MRSA, vancomycin-resistant *Enterococcus *, carbapenem-resistant *Acinetobacter baumannii* and *Pseudomonas aeruginosa* [53] (Fig. 5).

### Lessons from traditional medicine

How can associations between *Streptomyces* and traditional medical preparations help the search for new antibiotics?

### Silent antibiotic clusters

After whole-genome sequencing *Streptomyces coelicolor* A3(2), researchers discovered that instead of the usual two or three antibiotics detected under laboratory conditions, the genome encoded the potential to synthesize ten times this number [55]. This clearly means that there were other antibiotic-synthesis clusters that were not always expressed and are, therefore, now known as ‘silent clusters’ [56]. In recent years, it has been found that stress or extreme environmental triggers can stimulate these silent clusters to produce antibiotics [57–59]. Sometimes referred to as the ‘one strain, many compounds phenomenon’ (OSMAC) [60].

Many traditional medicines containing *Streptomyces* are associated with extreme environments typically low in nutrients, such as deserts, high altitudes, salt plains or cold areas where these bacteria form symbiotic associations with indigenous flora [30, 61]. These harsh conditions can also be used in the laboratory to awaken some silent gene clusters.

Extreme environments have also been investigated by researchers in the hope that *Streptomyces* from these areas would express a different repertoire of antibiotics. In Russia, researchers have been investigating the guts of amphipods inhabiting the bottom of Lake Baikal, where temperatures are rarely above 4 ˚C. They have isolated many *Streptomyces* strains that produce a new series of antibiotics effective against Gram-positive and Gram-negative bacteria [62, 63].

### Co-cultivation

It is quite typical to find multiple species of *Streptomyces* in traditional medical preparations such as soil or moonmilk [50, 54, 64]. Now, it has been discovered that competition and collaboration between *Streptomyces* and other species can also awaken silent antibiotic-synthesis clusters [65]. It has even been reported that the addition of *Streptomyces* that do not produce antibiotics can increase the antimicrobial potential of other antibiotic-producing *Streptomyces* through production of enhancement compounds like cyslabdan [66]. Although cyslabdan (known as a potentiator) has very little antimicrobial activity itself, it can enhance the antibiotic activity of β-lactams 1000-fold by inhibiting peptidoglycan synthesis in MRSA [66]. As a quicker route than co-culture, some researchers have been adding extracts of bacterial competitors or sub-inhibitory doses of antibiotics to elicit the production of antibiotics by silent gene clusters [58]. As discussed earlier, antibacterial resistance in *Streptomyces* is closely linked to antibacterial production [67].

### Multiple antibiotic therapy

Many traditional medicines contain a mixture of *Streptomyces* producing several antibiotics. This is a good strategy to reduce the possibility of resistance evolving quickly. The idea of using multiple antibiotics as a treatment option has become more widespread in the treatment of multi-resistant organisms and immunocompromised patients. The case of TB is one such example. Resistant TB strains started to appear not long after the introduction of streptomycin and isoniazid. The solution to this was to use an approach known as combination therapy. This is usually a combination of four second-line drugs (including amikacin, kanamycin, capreomycin and linezolid) with the addition of pyrazinamide over a period of 18–24 months. To add to this, two new anti-TB drugs, delamanid and bedaquiline, have also been approved for the first time in 50 years [68, 69].

### Media stimulation of antibiotic production from *Streptomyces*

The nutritional conditions under which *Streptomyces* are cultivated affects their antibiotic production. Many traditional medicines are applied in their raw state, usually incorporating some of the original isolation material, which can be chemically quite complex. Typical laboratory *Streptomyces* cultivation media contains a combination of yeast extract, complex starches, mannitol or some other sugar, humic acids on some occasions and perhaps supplementary minerals. These ingredients form the basis for the standard International *Streptomyces* project agars (ISP) 1–7 [70]. However, without some of the micronutrients or complex chemicals present in their original growth environments, some environmental antibiotic producers may lose their potency (antimicrobial production). To counteract this decline, recent innovations have seen researchers incorporate some native (isolation) material in their media, for instance soil that contains rare earth metals. These have been reported to stimulate some strains of *Streptomyces* to increase their antimicrobial production by 12-fold [71, 72]. Alternatively, other researchers have dispensed with intricate media formulations and tried to cultivate antibiotic-producing organisms *in situ* [73].

## Conclusion

Due to the increase in multi-resistant pathogens and the dwindling number of new products being approved for the health market, there is an urgent need to find new sources of antibiotics. In the last 80 years, *Streptomyces* has made a massive contribution to the field of medicine, not only through antibacterial antibiotics, but also through antifungal, antiparasitic and anticancer compounds. Recent isolations of *Streptomyces* from traditional medicine suggest that these bacteria have played an integral role in human health for longer than previously thought. This new source of *Streptomyces* can also help to replenish the much-depleted reservoir of emergency antibiotics to combat multi-resistant pathogens and perhaps provide the much-needed structural diversity needed for a new generation of novel antibiotics. Moreover, knowledge of their traditional use is more than a mere historical curiosity, as they could help us to unlock important factors in the complex production and/or application of antibiotics. Finally, to ensure the continued availability of this resource, it is imperative that the habitats and microbial genera associated with these *Streptomyces* are conserved, and that accurate information and data related to their prevalence, properties and characteristics are extensively documented.
